# Niche position and niche breadth effects on population abundances: A case study of New World Warblers (Parulidae)

**DOI:** 10.1002/ece3.11108

**Published:** 2024-03-17

**Authors:** Sandra Castaño‐Quintero, Julián Velasco, Alejandro González‐Voyer, Enrique Martínez‐Meyer, Carlos Yáñez‐Arenas

**Affiliations:** ^1^ Laboratorio de Ecología Geográfica, Unidad de Biología de la Conservación, Parque Científico y Tecnológico de Yucatán Unidad Académica Sisal ‐ Facultad de Ciencias, UNAM Chuburna Yucatan Mexico; ^2^ Posgrado en Ciencias Biológicas Universidad Nacional Autónoma de México Ciudad de México Mexico; ^3^ Instituto de Ciencias de la Atmósfera y Cambio Climático Universidad Nacional Autónoma de México (UNAM) Mexico City Mexico; ^4^ Departamento de Ecología Evolutiva, Instituto de Ecología Universidad Nacional Autónoma de México (UNAM) Mexico City Mexico; ^5^ Departamento de Zoología, Instituto de Biología Universidad Nacional Autónoma de México (UNAM) Mexico City Mexico

**Keywords:** ecological niche, niche breadth, niche position, population abundance

## Abstract

Species abundance patterns are influenced by a myriad of factors, including habitat availability and ecological niche characteristics. However, the evidence concerning the specific impact factors such as niche position and niche breadth on mean and maximum abundances in vertebrates at a broad geographical scale remains inconclusive. In this study, we investigated the influence of niche position and breadth on the abundance of 47 species of birds belonging to the Parulidae family, commonly known as New World Warblers. We obtained data on abundance and presence records spanning the reproductive distribution of these species and employed the outlying mean index analysis to calculate niche position and niche breadth. We assessed the relationship between abundance metrics and niche descriptors using phylogenetic regressions to account for the non‐independence resulting from phylogenetic ancestry. Initially, we developed individual models for each predictor and subsequently formulated a multi‐predictor model encompassing niche position, niche breadth, and their interaction. Our findings revealed a negative relationship between niche position and both mean and maximum abundance, while niche breadth exhibited a positive relationship with these niche characteristics. Notably, the results of the multi‐predictor models indicated that niche position exerted the most substantial influence on both mean and maximum abundance. Additionally, the interaction between niche position and niche breadth had the most positive and significant contribution to mean population abundance. This study underscores the need for future research in other vertebrates to delve into the mechanisms underlying these patterns. Such endeavors will not only enhance our understanding of ecological dynamics but also equip us with predictive capabilities to anticipate population responses to environmental changes effectively.

## Graphical abstract

We investigated the influence of niche position and breadth on the abundance of 47 species of birds belonging to the Parulidae family, commonly known as New World Warblers. Our findings revealed a negative relationship between niche position and both mean and maximum abundance, while niche breadth exhibited a positive relationship with these niche characteristics. The interaction between niche position and niche breadth had the most positive and significant contribution to mean population abundance.

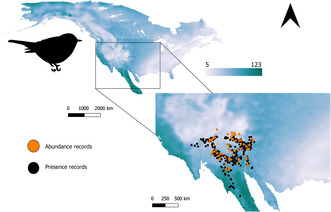

## 1 INTRODUCTION

Understanding the geographic distribution of species' population abundances, ranging from high to low population densities, has been a fascinating topic of researchers (Brown, 1984; Gaston, 1994; Sagarin & Gaines, 2002). This has led to the development of the abundance‐centre hypothesis, which applies to both geographic and ecological space. Initially proposed in relation to geographic space, the abundance center hypothesis suggests that within a species' distribution area, population abundances are higher near the center and gradually decrease toward the boundaries (Brown, 1984; Brown et al., 1995). From an ecological perspective, the abundant‐niche‐centre hypothesis is rooted in the ecological niche concept, which states that a species' fundamental niche exists within a multidimensional abstract space with as many axes as there are environmental states that allow a species to exist (Hutchinson, 1957). The abundant niche‐center hypothesis extends this concept, proposing that spatial abundance patterns are influenced by the spatial arrangement of environmental conditions relative to a species' niche centroid across its distribution range (Martínez‐Meyer et al., 2013; Yañez‐Arenas et al., 2012), although this is a controversial issue (Jiménez‐Valverde et al., 2020). For instance, this pattern may be constrained by factors such as biotic interactions and/or mobility limitations (Soberon & Peterson, 2005).

Within community ecology, ecological niches are often characterized by two key dimensions: niche position (NP) and niche breadth (NB). Niche position, which aligns with the abundance‐center hypothesis, quantifies the distance between a species' average environmental conditions and the average conditions available in a given environmental space. This concept suggests that species utilizing the most common environments will tend to have higher abundances than those favoring rarer environments (Doledec et al., 2000; Hanski, 1993). For instance, species with niche positions that are closer to the average environmental conditions are more likely to find suitable habitats (Venier & Fahrig, 1996). Invoking the abundant niche‐center hypothesis, if population abundance reflects environmental combinations and is relative to an environmental optimum (niche centroid), then species with more frequent environmental optima in a given region will have higher population sizes than species with marginal or scarcely available environmental optima. Conversely, niche breadth refers to the range of environmental conditions tolerated by a species, and predicts that species with broader tolerances will exhibit higher abundances (Brown, 1984). Greater niche breadth increases the likelihood of encountering suitable conditions within a given area. When considering the internal structure of the niche and its relationship with available environments, niche position (NP) combined with niche breadth (NB) could help explaining both environmental and spatial abundance patterns.

Until now, a series of studies have explored the effects of NP and NB on population abundances. However, these studies have often been constrained by specific factors such as limited study areas (but see Yancovitch Shalom et al., 2020), data scarcity, or the inclusion of species phylogenetically unrelated (Heino & Grönroos, 2014; Vela‐Díaz et al., 2020; Wen et al., 2021). Within these studies, contrasting results have emerged. For instance, studies focusing on tree species have revealed that higher abundances and occupancy are often concentrated in non‐marginal environments, with NB exerting minimal influence on mean abundances and occupancy (Vela‐Díaz et al., 2020). Conversely, in a study of mammal species along an elevation gradient, NB had a positive effect on mean abundance, while NP had a negative relationship with abundance. Populations tend to exhibit lower abundances as they move farther from the mean environments compared to the available environments (Wen et al., 2021). When it comes to birds, contrasting results have been found. One of the most influential variables on the density of birds from an island in the Canary archipelago was habitat breadth, which, similar to NB, measures the variety of vegetation structure categories where the species was found (Carrascal et al., 2015). When studying British birds, researchers found that species with wider niche breadths did not exhibit more abundant populations compared to species with narrower niches. Support was found only for the hypothesis related to NP, where species with marginal NP values had lower abundances than those with central NP values. In other words, these findings suggest that species with mean tolerances overlapping with atypical environments occurred at lower abundances (Gregory & Gaston, 2000). Similarly, in stream invertebrates, NP explained only a small amount of local abundance, while NB did not contribute to explaining local abundance (Heino & Grönroos, 2014). Studies centered on diatoms and insects showed that NP has a negative correlation with mean local abundance, while NB was not a significant predictor (Rocha et al., 2018). The mixed results of the NB and NP effect may be due to various factors, including their interaction. For example, species with central NP may have high abundances, even if they have narrow NB. However, species with broad NB but marginal NP may exhibit abundances that are affected by both NP and NB in a complex way, with a particular statistical relationship and direction. To gain a better understanding of the individual and combined effects of NP and NB on population abundances, it is important to consider large sample sizes and abundance data derived from systematic samplings. These samplings should encompass broad temporal and geographical scales and include a well‐represented phylogenetic group.

In this study, we investigate the effects of niche position and breadth on the abundance of migratory species within the Parulidae family, the New World Warblers. We expect that both mean and maximum abundances will decrease as the distance from mean climates increases, indicating higher environmental marginality. Conversely, we anticipate that both mean and maximum abundances will be higher in the proximity of the most common climates. Additionally, we expect that species with broader niche breadths will exhibit greater mean and maximum abundances compared to species with narrower niche breadths. Finally, we hypothesize that the combined effect of niche breadth and position will show a compensatory pattern, where the direction of the statistical relationship depends on the specific niche characteristic that has a stronger individual effect.

## 2 MATERIALS AND METHODS

### 2.1 Study area and environmental variables

The study area encompasses a subset of North America. Specifically, we delineated a polygon by combining the breeding season distribution areas of Parulidae species for which abundance data were available. The distribution polygons for each species were obtained from BirdLife International (BirdLife International & the Handbook of the Birds of the World, 2022).

To characterize the environmental conditions within the study area, we used the CHELSA climatic database (Karger et al., 2017) at a 10° resolution, which is appropriate given the extent of our study area and the spatial precision of the georeferenced abundance data (see below) (Dornak et al., 2013; Osorio‐Olvera et al., 2020). CHELSA was developed by applying a statistical downscaling process to satellite data, resulting in a more accurate representation of climate variables (Karger et al., 2017). We downloaded 19 climate variables derived from monthly precipitation and temperature values, which capture average trends, seasonality, and factors that limit species' distributions. To reduce multicollinearity among the environmental variables, we performed a variance inflation factor (VIF) analysis. Furthermore, we carried out a predictor selection process where we employed the ellipsoid_selection function from the ntbox package (Osorio‐Olvera et al., 2016). This function facilitates variable selection based on omission rates in environmental space E. We constructed our models using 95% of the refined presence data and evaluated them using abundance information. We opted for the minimum volume ellipsoid methodology because theoretical and physiological evidence suggests that species' ecological niches exhibit convex shapes (Angilletta, 2009; Hooper et al., 2008; Maguire, 1973; Soberón & Nakamura, 2009). Convex ecological niches can manifest as ellipsoids or polyhedra, with the highest environmental suitability located near their centroids (Maguire, 1973; Martínez‐Meyer et al., 2013). Empirical findings support the notion that regions with conditions closely resembling the niche centroid exhibit the highest population abundance (Martínez‐Meyer et al., 2013; Yañez‐Arenas et al., 2012), as well as greater genetic diversity (Lira‐Noriega & Manthey, 2014). The algorithm selects the best models and associated predictors by evaluating the omission rate in environmental space for each predictor combination. The results are then ranked by omission rate, with the lowest omission rate indicating the best model. Based on the VIF results, information from the literature, and the predictor selection process, we selected mean annual temperature (Bio1), annual precipitation (Bio12), and precipitation seasonality (Bio15) as the key environmental variables for further analysis.

### 2.2 Abundance and presence records

Abundance data were downloaded from the North American Breeding Bird Survey (BBS; https://www.pwrc.usgs.gov/bbs/), a large‐scale spatial and temporal bird monitoring program. Initiated in 1966, the BBS tracks the population status and trends of North American birds and annually provides an index of population abundance, as well as raw sampling data. Each sampling route is approximately 50 km long with count points located 1 km apart, where each count point is surveyed for 3 min. We downloaded the abundance count tables and route metadata for the period between 1966 and 2021 and averaged the counts for each route (Figure S1). The analysis included 47 species from the Parulidae family for which data were available from at least 19 routes. As a measure of species abundance, we estimated the average counts across all the years in which a route was sampled (Osorio‐Olvera et al., 2020). We obtained mean and maximum abundance estimates for each species for further analysis.

**FIGURE 1:**
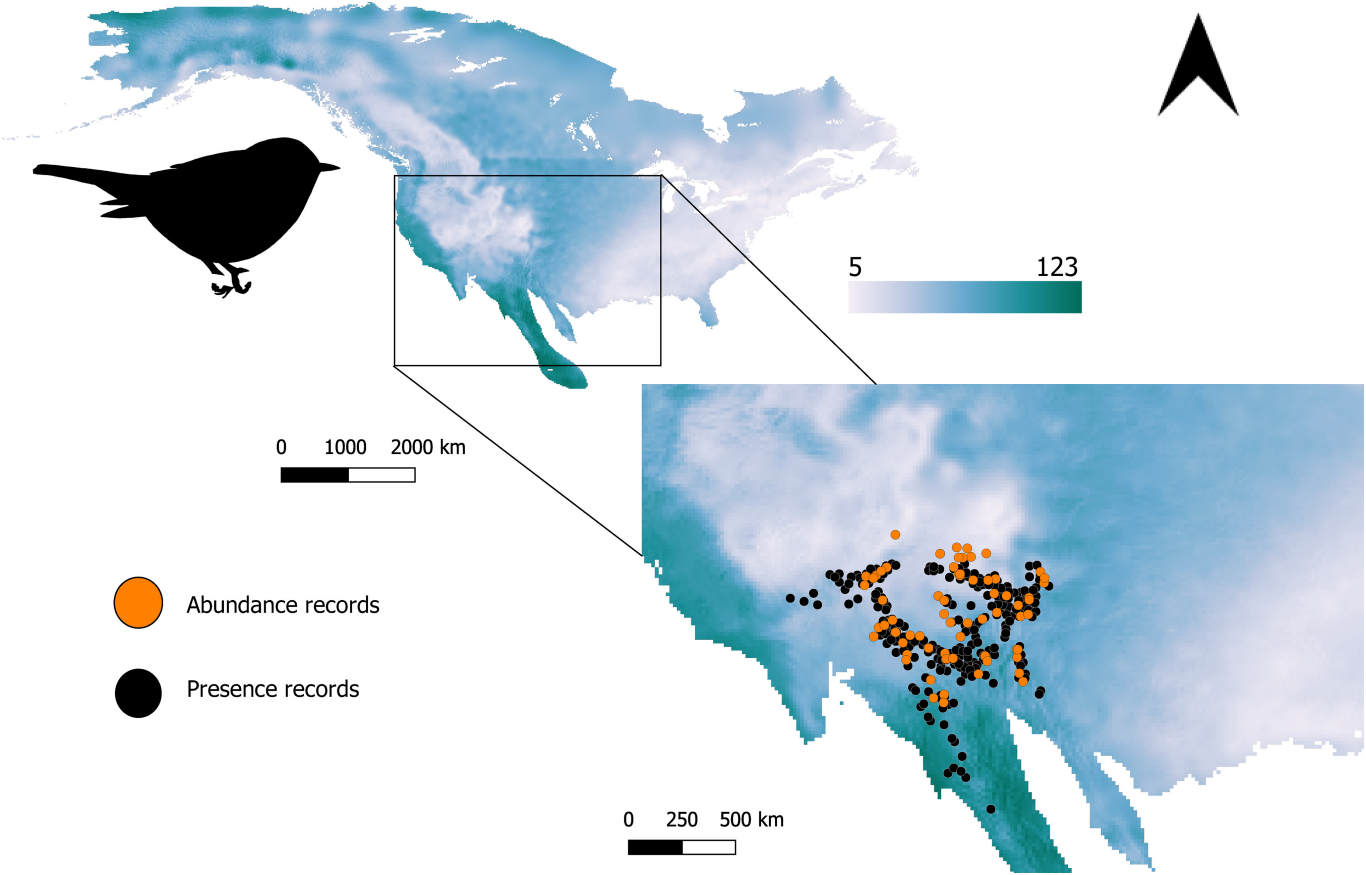
Study area and abundance and presence records for *Setophaga gracie* along the precipitation seasonality gradient. *Setophaga* silhouette downloaded from phylopic.org.

Presence data for each species were downloaded from the global biodiversity information facility (GBIF; gbif.org). Presence records were filtered to retain only those recorded during the breeding months: May to August. The data were then cleaned by removing poorly georeferenced and duplicate presence records and retaining only those that intersected with the reported breeding areas by BirdLife International. Figure 1 shows abundance and presence records for *Setophaga gracie* along a portion of the study area with the precipitation seasonality gradient, as an example of the data available for each species.

### 2.3 Niche position and niche breadth estimation

Niche position (NP) and niche breadth (NB) were determined for each species using the outlying mean index (OMI) analysis, as described by Doledec et al., 2000. The OMI method uses a combination of principal component analysis (PCA) on the environmental conditions within the study area and a table of presence records for each species. This multivariate method leverages environmental variables to place species along relevant axes, without imposing constraints on their response curves. It also balances the influence of species‐rich and species‐poor sites. As a result, the analysis generates a mean niche position (defined as the distance between a species' mean environmental conditions and the study area's mean for each gradient) in each selected gradient (niche position) for each species (Thuiller et al., 2005). To maintain independence between the abundance and presence datasets, here we used only presence records from GBIF. The OMI analysis was performed using the ade4 package in R (Dray & Dufour, 2007; R Core Team, 2022).

### 2.4 Statistical analysis

To investigate the relationship between niche descriptors and abundance metrics, we applied phylogenetic generalized least squares regression (PGLS) with a Lambda correlation structure. This method accounts for the non‐independence of data due to shared evolutionary history among species by incorporating an error matrix based on a phylogeny (Martins & Hansen, 1997). To do this, we downloaded the Parulidae family phylogeny published by Lovette et al. (2010), which used a RaxML maximum likelihood analysis to estimate a fully resolved topology for 110 species. We retained only the branches corresponding to the species included in this study.

We ran models individually for each predictor variable and then constructed models with all predictors and their interactions (multi‐predictor PGLS models). Additionally, we included three control variables: the percentage of the species' distribution area that overlapped with the countries where the BBS operates, body mass, and the sample size of species abundance. The PGLS analyses were conducted using the gls function from the nlme (Pinheiro et al., 2021) package in R.

Furthermore, we applied automated model selection using the size‐corrected Akaike information criterion (AICc) with the MuMln package (Barton, 2020), which ranks models based on their AICc values. We sought to identify the most informative multi‐predictor PGLS models, prioritizing those with delta AICc values below two. As all subsequent models for mean abundance exhibited delta AICc values exceeding 2, we solely report the model with the lowest AICc (Table S1). However, for maximum abundance, we focus on the second‐best model specifically because it includes all predictors' interactions, potentially capturing relevant complexity, and its AICc is within 2 units of the best (Table S1). Finally, we performed an ANOVA in R on the best mean abundance model and the best maximum abundance model.

## 3 RESULTS

**FIGURE 2:**
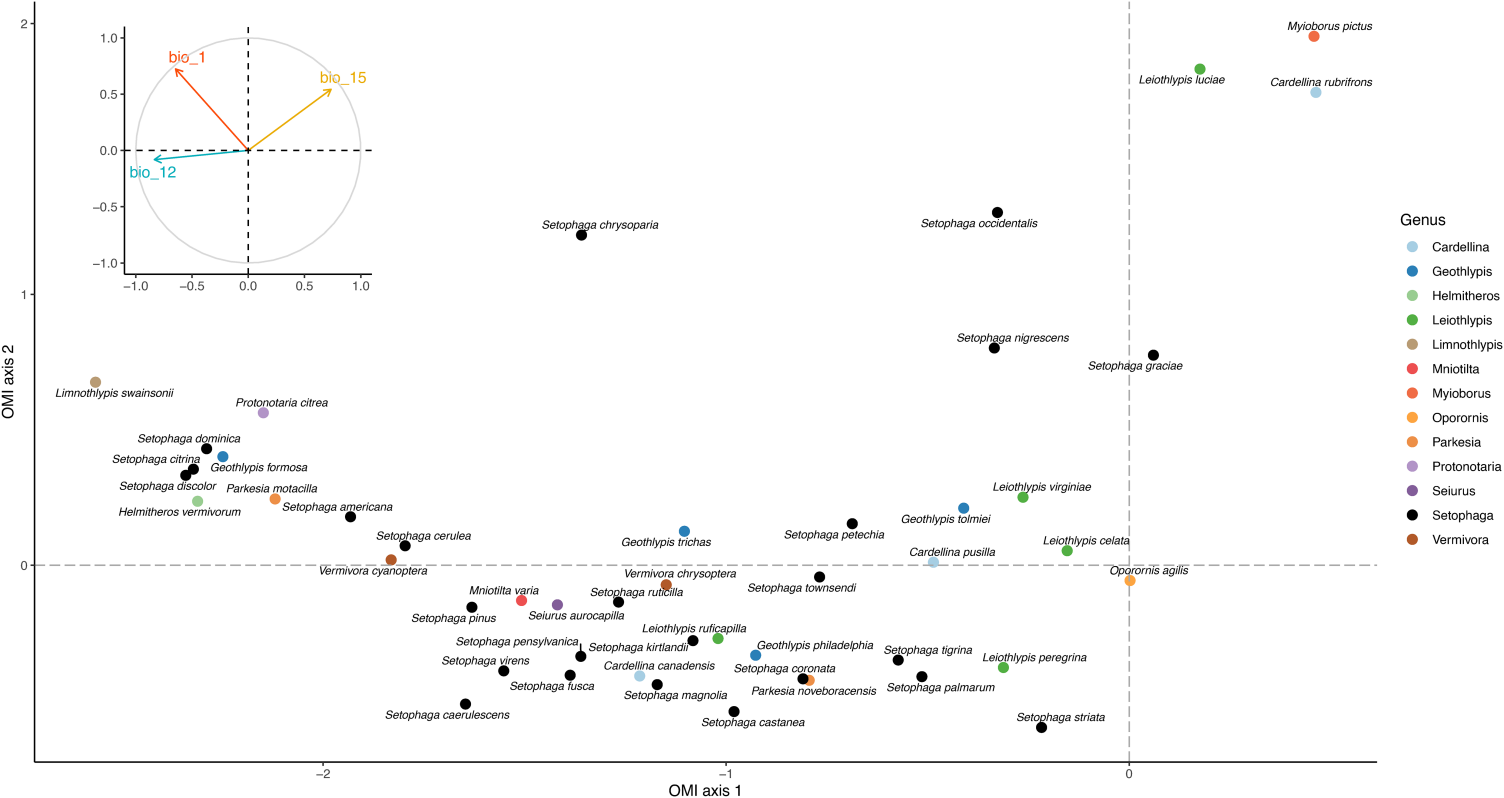
Niche position of species in the Parulidae family along the OMI axes. The colors represent the genera to which each species belongs. In the top left corner, the environmental predictors are displayed with respect to the axes. Bio1: annual mean temperature, Bio12: annual precipitation amount, and Bio15: precipitation seasonality.

The first two axes of the OMI analysis explained 82.7% of the climatic variation observed in the Parulidae species. The first axis accounted for 55.2% and the second axis for 27.5%. The temperature variable had the greatest influence on the OMI axes, followed by precipitation seasonality. This analysis suggests that Parulidae species are primarily associated with the annual mean temperature and annual precipitation. Only four species, from different genera, were found to occupy the quadrant associated with precipitation seasonality (Figure 2).

**FIGURE 3:**
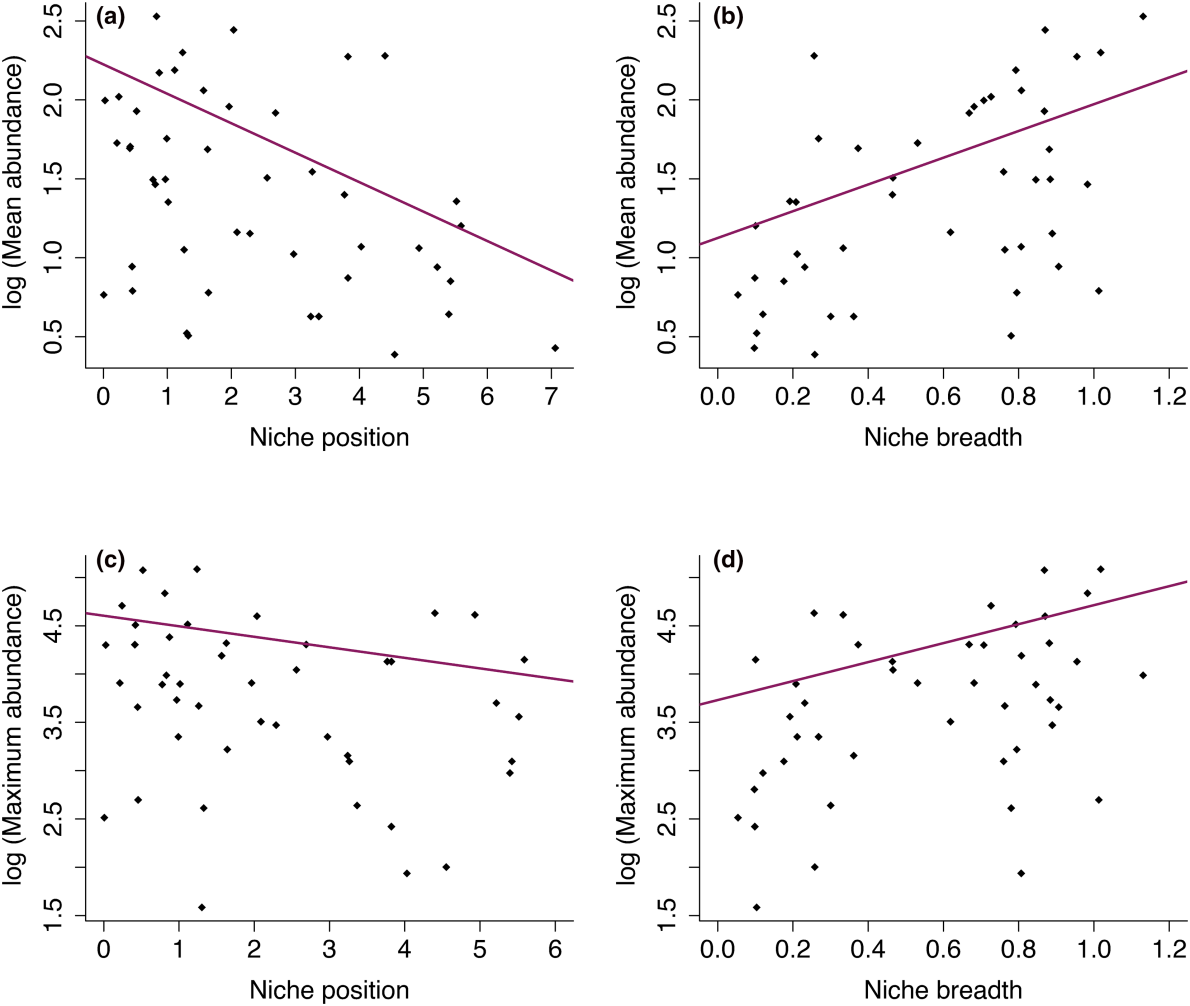
Relationship between niche position, niche breadth, and abundance metrics of some parulidae family species. Upper panel: mean abundance relationship with NP (a) and NB (b). Lower panel: maximum abundance relationship with NP (c) and NB (d).

**FIGURE 4:**
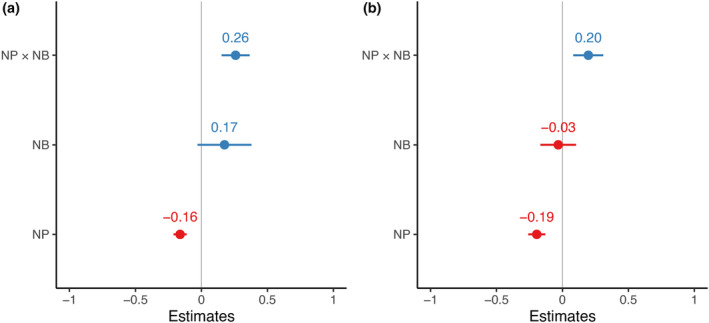
PGLS model estimates per abundance metric regressed against niche characteristics and their 95% confidence intervals. (a) Predictor contribution to mean abundance. (b) Predictor contribution to maximum abundance. NP × NB represents the interaction between niche position (NP) and niche breadth (NB).

Individual PGLS analyses revealed that species with marginal niche position (NP), indicating greater deviation from the mean conditions available, had lower mean and maximum abundance (Figure 3). Similarly, species with wider NB values showed higher mean and maximum abundance. The multi‐predictor PGLS models consistently showed that NP had a significant negative effect on both mean and maximum abundance, while NB had a non‐significant effect (Figure 4, Table 1). However, considering the interaction between NP and NB revealed a stronger positive effect on mean and maximum abundances. The confidence intervals supported the effect of the interaction between niche characteristics on mean and maximum abundance, as well as the individual effect of NP on both abundance metrics (Figure 4).

**TABLE 1:** Estimates, 95% confidence intervals, and *p*‐value of multi‐predictor PGLS models.

	log (Mean abundance)			log (Maximum abundance)		
	*λ* = 0.912			*λ* = 1.031		
Predictors	Estimates	CI	*p*	Estimates	CI	*p*
(Intercept)	1.72	1.22 to 2.22	**<0.001**	4.56	4.03 to 5.08	**<0.001**
NP	−0.16	−0.26 to −0.06	**0.002**	−0.19	4.03 to 5.08	**0.004**
NB	0.17	−0.24 to 0.59	0.396	−0.03	−0.30 to 0.24	0.813
NP*NB	0.26	−0.24 to 0.59	**0.018**	0.2	−0.30 to 0.24	0.091

*Note*: Bold values indicate statistically significant results (*p* < .05).

Abbreviations: CI, confidence intervals; *p*, *p*‐value.

**FIGURE 5:**
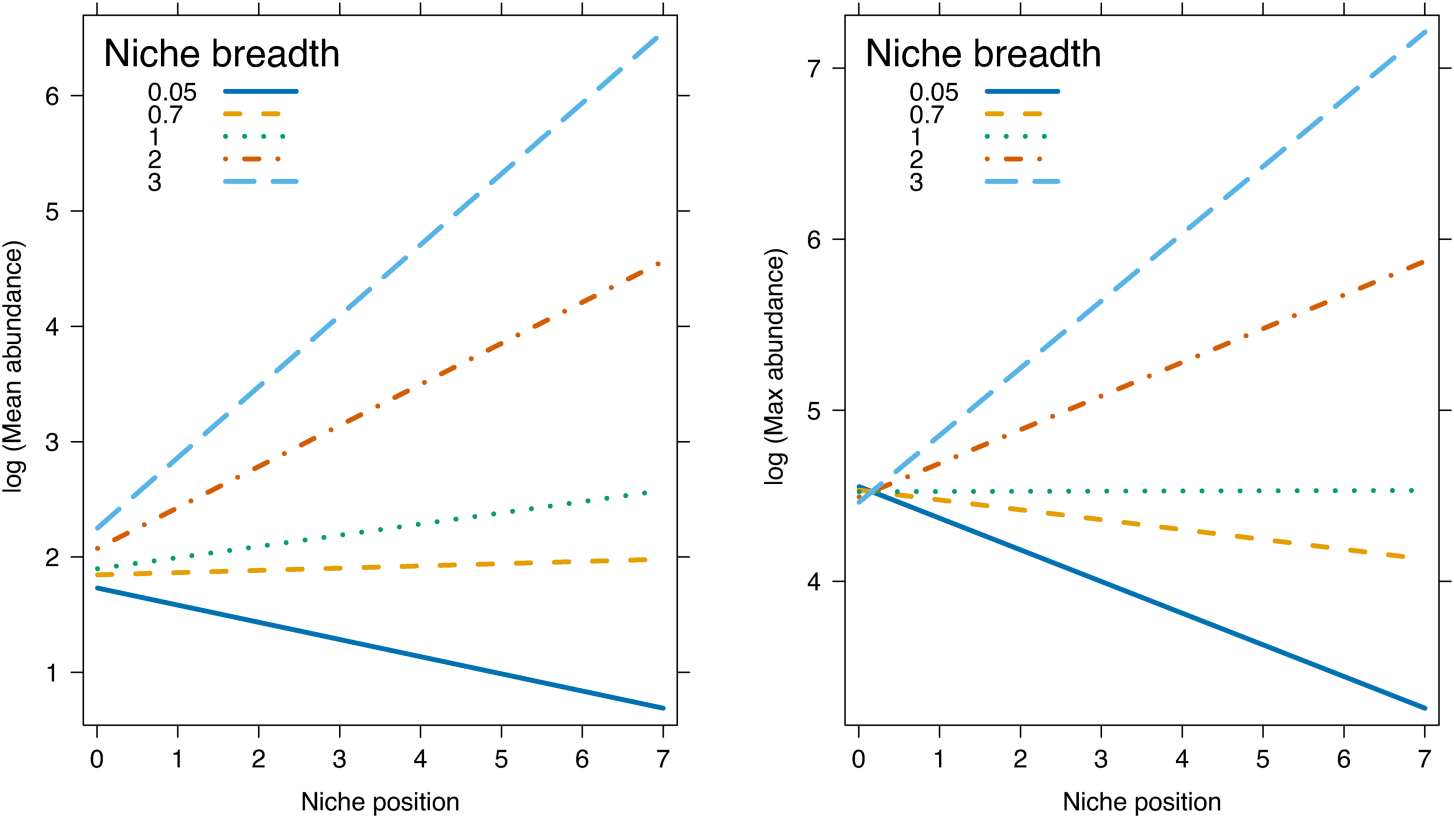
Interaction plot of niche position (NP) and niche breadth (NB) where the effect of NP on abundance metrics is evaluated for different NB values.

We identified a threshold in the interaction between niche position and niche breadth, where NP exhibits the expected negative relationship with abundance metrics if NB is small. As NB increases, NP loses its negative relationship with abundance metrics (Figure 5). Finally, the ANOVA results showed that all of the predictors in the multi‐predictor PGLS model, including the interaction term between NP and NB, had a significant effect on average abundance. In contrast, only NP had a significant effect on maximum abundance (Table 2).

**TABLE 2:** ANOVA of multipredictor PGLS models.

	log (Mean abundance)			log (Maximum abundance)		
	numDF	*F*‐value	*p*‐value	numDF	*F*‐value	*p*‐value
(Intercept)	1	337.252067	5.70E‐22	1	1.52637E+14	1.79E‐271
NP	1	38.0373838	2.09E‐07	1	23.54041572	1.64E‐05
NB	1	4.745976	.03489536	1	1.274630791	.26515908
NP × NB	1	6.04507942	.01804735	1	2.990156343	.09094622

Abbreviation: numDF, numerator degrees of freedom.

## 4 DISCUSSION

Our findings provide strong support for the proposed hypotheses at least regarding the variables used in this study, demonstrating a negative relationship between NP and both the mean and maximum abundance values, while showing a positive relationship with NB. Furthermore, the combined positive interaction of these characteristics had the greatest contribution to abundances. To the best of our knowledge, this is the first evaluation of the effects of NP and NB on abundances in a phylogenetic context and broad spatial scale within a group of terrestrial vertebrates. These findings suggest that niche position and niche breadth are important factors that influence the abundance of terrestrial vertebrate species.

Niche position has been identified as a determining factor not only in population abundances but also in population vital rates and genetic diversity. For instance, populations of Freshwater pearl mussels residing in suboptimal environments rarely experience recruitment. This implies a higher potential risk of extinction for these populations (Tamario et al., 2022). The ability of species to occupy the most available environments, particularly those near the most common environments (referred to as NP), has been a key factor in explaining the abundance and occupancy patterns of bromeliad invertebrates at a large spatial scale (Marino et al., 2020). However, our results show that although there is a negative relationship between niche position and abundance, NP contributes positively to abundance when it interacts with niche breadth in multipredictor‐PGLS models. It has been suggested that in terrestrial species, their physiological or behavioral capacity to regulate temperature makes them less susceptible to reduced performance in suboptimal environments (Waldock et al., 2019). Furthermore, it is possible that the optima of their ecological niches, which may not necessarily coincide with the most available environments, have a greater influence than the most common environments. For example, in birds from North Africa and North America, population abundance followed the pattern of environmental suitability, suggesting that the depletion or reduction of suitable areas restricts the presence of bird populations (Osorio‐Olvera et al., 2020; Tellería et al., 2021).

On the other hand, species abundance may decrease from a point of maximum abundance that does not necessarily coincide with the optimal environmental conditions for the species or for the most common environments. This phenomenon is referred to as the abundant core (Fristoe et al., 2023). The abundant core is a region within the species' range where maximum abundances are concentrated, with abundances decreasing from this point (Fristoe et al., 2023). Biotic interactions, such as competition, predation, and parasitism, have been proposed as potential factors that can shift maximum abundances away from the optimal range of environmental conditions (Yancovitch Shalom et al., 2020). Additionally, other factors such as dispersal and the Allee effect, have been suggested as mechanisms that can weaken the expected patterns of the abundant center (Feng & Qiao, 2022; Osorio‐Olvera et al., 2019).

Furthermore, it has been reported that certain species of trees in forest communities exhibit population abundances that are negatively related to NP. However, this relationship depends on the specific geographic location of the forest community that is being studied (Vela‐Díaz et al., 2020). This result is explained as the outcome of the effect of unmeasured biotic and abiotic variables that are still relevant within the *n*‐dimensional niche of species (Chase & Leibold, 2009; Hutchinson, 1957; Siqueira et al., 2009; Vela‐Díaz et al., 2020).

For the Parulidae species that were evaluated in our study, we found that niche breadth did not make a significant contribution to mean or maximum abundance in the multi‐predictor models, but had a significant effect according to the ANOVA. This could happen when the predictor interacts with another predictor in the model, such as niche position. Moreover, research has shown that some bird species inhabiting a larger number of habitats may be associated with a wider range of environmental variation but, due to other biological factors not included, may exhibit population sizes lower than expected (Carrascal et al., 2015). In other organisms, NB has been identified as a better predictor of abundance than niche position (Siqueira et al., 2009). However, for some insects, it has shown limited importance (Heino, 2005) or has not contributed significantly to explaining the variation in the abundance of aquatic invertebrates (Heino & Grönroos, 2014).

The mixed results regarding the effect of NB on abundance may be attributed to its interaction with niche position and its additive effect on population abundance. If a species with a wide NB prefers common environments and, as a result, has a central NP, the latter will contribute more to explaining abundance patterns. Niche position will also be more influential when a species, possessing a low NB, prefers or specializes in common environments, indicating a central NP. There should indeed exist a balance between these niche characteristics that explains why the interaction of NP and NB, in addition to being positive, contributed more to explaining the mean and maximum abundances of the evaluated birds in this study. It is possible that species with a wide NB can occupy both common and less common environments, experiencing a decline in abundance towards their suboptimal tolerances after reaching a threshold between NP and NB.

The results presented here map the existence of the same patterns of niche position and niche breadth influencing abundance that have previously observed at smaller scales in plants and invertebrates, but now in a group of vertebrates. Our study model is appropriate because migratory birds do not have strict dispersal limits, which is significant because the environments they choose to explore align with their physiological tolerances, enabling the models to accurately capture patterns of physiological responses (Owens et al., 2013). Nonetheless, a great deal of variation in abundance is still not explained by the factors that we included in our models. The above could be due to several factors, including: (i) biotic and abiotic predictors that affect abundance were not taken into account; (ii) only linear regressions were applied, allowing for the inclusion of the correlation structure given by phylogeny. However, it would be worthwhile to explore other types of statistical relationships while attempting to account for the non‐independence of species due to their common evolutionary history; (iii) the coarse spatial scale we used may underestimate variability that could exist at finer scales, potentially compromising the level of detail when calculating NP and NB; (iv) we only had abundance data for 47 of ~105 species in the Parulidae family. Perhaps by adding population information for other species, we can provide more precise support for the patterns found or even discover more variation. Similarly, it would be valuable to test the generality of these patterns in other groups of vertebrates to approximate the underlying mechanisms and determine whether tropical species exhibit different patterns than temperate species.

In summary, we found the expected relationships between niche characteristics and abundance metrics, with the strongest positive contribution to mean abundance from the interaction of the two niche characteristics evaluated. This suggests the existence of a balance between niche position and niche breadth that acts on population abundance, at least for this group of species. However, further research on these niche characteristics and their effects on abundance is necessary to propose mechanisms that can help us to understand why niche position and niche breadth have the effects that they do on abundance and assess whether the same patterns that we observed in this study of New World warblers are also observed in other groups of vertebrates.

## AUTHOR CONTRIBUTIONS

**Sandra Castaño‐Quintero:** Conceptualization (equal); data curation (lead); formal analysis (lead); investigation (lead); methodology (equal); visualization (lead); writing – original draft (lead); writing – review and editing (lead). **Julián Velasco:** Conceptualization (equal); formal analysis (equal); methodology (equal); supervision (equal); validation (equal); writing – original draft (supporting); writing – review and editing (supporting). **Alejandro González‐Voyer:** Conceptualization (equal); methodology (equal); validation (equal); writing – review and editing (supporting). **Enrique Martínez‐Meyer:** Conceptualization (equal); supervision (equal); validation (equal); writing – review and editing (supporting). **Carlos Yáñez‐Arenas:** Conceptualization (equal); supervision (equal); validation (equal); writing – original draft (supporting); writing – review and editing (supporting).

## Supporting information


Figure S1.

Table S1.

## Data Availability

The data that support the findings of this study are openly available in the FigShare Data Repository: https://figshare.com/s/c71a49da600ceed9bc53.
