# Supplementary figures and images for: Niche position and niche breadth effects on population abundances: A case study of New World Warblers (Parulidae)
Source: Ecol Evol. 2024 Mar 17;14(3):e11108. doi: 10.1002/ece3.11108 (PMC10944703; doi:10.1002/ece3.11108)

● Routes

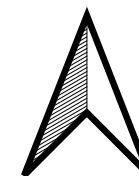

0 1,000 2,000 km

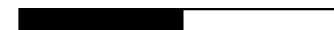

Supplement: Supplementary file 1 — Figure S1. [file ECE3-14-e11108-s001.pdf]
